# Single Nucleotide Polymorphisms of Toll-Like Receptor 4 Decrease the Risk of Development of Hepatocellular Carcinoma

**DOI:** 10.1371/journal.pone.0019466

**Published:** 2011-04-29

**Authors:** Shi Minmin, Xu Xiaoqian, Chen Hao, Shen Baiyong, Deng Xiaxing, Xie Junjie, Zhan Xi, Zhao Jianquan, Jiang Songyao

**Affiliations:** 1 Research Institute of Digestive Surgery, Ruijin Hospital, Shanghai Jiao Tong University School of Medicine, Shanghai, People's Republic of China; 2 Department of Hematology, Changhai Hospital, Second Military Medical University, Shanghai, People's Republic of China; 3 Department of Surgery, Ruijin Hospital, Shanghai Jiaoton University School of Medicine, Shanghai, People's Republic of China; Institut Pasteur, France

## Abstract

**Background:**

Toll-like receptor 4 (TLR4) is a key innate immunity receptor that initiates an inflammatory response. Growing evidence suggests that mutation of TLR4 gene may play a role in the development of cancers. This study aimed to investigate the temporal relationship of single nucleotide polymorphisms of TLR4 and the risk of hepatocellular carcinoma, a single center-based case-control study was conducted.

**Methods:**

A systematic genetic analysis of sequence variants of TLR4 by evaluating ten single-nucleotide polymorphisms was performed from 216 hepatocellular carcinoma cases and 228 controls.

**Results:**

Six single nucleotide polymorphisms of the TLR4 in the 5′-untranslated region and intron were associated with risk of hepatocellular carcinoma. Individuals carrying the heterozygous genotypes for the rs10759930, rs2737190, rs10116253, rs1927914, rs12377632 and rs1927911 had significantly decreased risk of hepatocellular carcinoma (adjusted odds ratio [OR], from 0.527 to 0.578, *P*<0.01) comparing with those carrying wild-type homozygous genotypes. In haplotype analysis, one haplotype (GCCCTTAG) of TLR4 was associated significantly with decrease of the occurrence of hepatocellular carcinoma (OR, 0.556, 95% confidence interval [CI], 0.407–0.758, *P* = 0.000).

**Conclusions:**

Collectively, these results suggested that the risk of hepatocellular carcinoma was associated with TLR4 sequence variation. TLR4 single nucleotide polymorphisms may play an important protective role in the development of hepatocellular carcinoma.

## Introduction

Toll-like receptors (TLRs) are a group of highly conserved molecules that allow the immune system to sense molecules that are present in most classes of pathogens. The recognition of pathogen associated molecular patterns (PAMPs) by TLRs is a cornerstone of innate immunity and provides a quick and highly efficient response to pathogens. Much of the evidence supports a role for TLR-mediated signaling in inflammation. TLR4 is located on chromosome 9, and exposure to bacterial products or pro-inflammatory cytokines increases TLR4 expression in monocytes and polymorphonuclear leukocytes [1]. The occurrence of TLR4 activation is confirmed during the systemic inflammatory response [2]–[4]. TLR4-deficient mice showed lower response to viral and bacterial infection than did wild-type mice [5], which suggested that TLR4 activation pathway can initiate innate immune responses to both bacterial and viral pathogens.

Emerging evidence from association investigations has shown that TLR4 polymorphisms are associated with chronic and recurrent inflammation and the occurrence of related cancer [6]. Recently, several studies showed that variants in TLR4 were related with the risk of prostate cancer in Eastern Asian population [7] or Western population [8],[9]. In other studies, TLR4 polymorphism increases risk of gastric carcinoma [10] and its precursors [11]. However, there has not been any investigation about TLR4 polymorphisms in hepatocellular carcinoma yet. Healthy liver contains low gene transcription of TLR4 and its adaptor molecules such as CD14, myeloid differentiation protein-2 (MD-2) and myeloid differentiation factor 88 (MyD88) [12]. However, under pathologic conditions, TLR4 activates inflammatory-signaling pathways in the liver and is actively involved in the pathophysiology in some hepatic diseases including Hepatitis B virus (HBV) [13]. HBV is a DNA virus that causes an acute hepatitis, which is self-limited in 80–90% of adults but becomes chronic in the remaining 10–20% of adult patients. Chronic hepatitis B is the leading cause of hepatic cirrhosis and hepatocellular carcinoma [14]. Hepatocellular carcinoma is the fifth most common cancer worldwide and the third leading cause of cancer-related death, with more than half the cases occurring in China [15]. Notably, most hepatocellular carcinoma cases in China are associated with chronic hepatitis B virus (HBV) infection [16].

In light of the strong biological support for a role for TLR4 in carcinogenesis and the potential importance of inflammation and inflammatory genes in hepatocellular carcinoma development, we hypothesized that single-nucleotide polymorphisms of TLR4 are associated with hepatocellular carcinoma susceptibility. To test this hypothesis, we performed a systematic genetic analysis in a Chinese population hospital-based hepatocellular carcinoma case-control study.

## Results

The selected characteristics of the hepatocellular carcinoma cases and control subjects are summarized in Table 1. There were no significant differences between patients with hepatocellular carcinoma and controls in terms of age, sex and HBV carriers (*P*>0.05). Among hepatocellular carcinoma cases, α-FP level higher than 400 ng/ml was in 33.1% of patients and α-FP below 400 ng/ml was in 66.9%. For UICC classification, 45.83% were in tumor stage I to II and 54.17% in stage III–IV.

**Table 1:** Baseline characteristics of hepatocellular carcinoma cases and controls.

	Cases n (%)	Controls n (%)	*P* Value
Number	216	228	
Mean age (yr)	54.3±12.4	47.2±13.7	
Age group			
≤55	123 (56.94%)	153 (67.11%)	0.299
>55	93 (40.06%)	75 (32.89%)	
Sex			
Male	182 (84.26%)	184 (80.7%)	0.273
Female	33 (15.74%)	44 (19.3%)	
HBV carriers			
Yes	177 (81.94%)	181 (77.2%)	0.074
No	39 (18.06%)	47 (22.8%)	
α-FP level			
>400 ng/ml	82 (33.1%)	NA	
<400 ng/ml	134 (66.9%)		
UICC classification			
Stage I–II	99 (45.83%)	NA	
Stage III–IV	117 (54.17%)		

α-FP: alpha-fetoprotein; NA: no data.

The observed genotype frequencies of all 10 polymorphisms in both of patients and controls conformed to the Hardy-Weinberg equilibrium (all *P*>0.05). The distributions of genotypes for TLR4 were shown in Table 2. Chi Square analysis of the genotypes revealed significantly different distributions in six TLR4 SNPs (rs10759930, rs2737190, rs10116253, rs1927914, rs10759932 and rs1927911) between the group with hepatocellular carcinoma and the control group (*P*<0.05). Among them, five SNPs were located in the 5′-UTR of TLR4 gene while one SNP was located in the region of intron.

**Table 2:** Genotype frequencies of TLR4 single nucleotide polymorphisms.

SNP	Location	Genotype	Cases, n (%)	Controls, n (%)	*P* Value
rs10759930	5′-UTR	TT	90 (41.67)	70 (30.70)	0.039
		CT	89 (41.20)	118 (51.75)	
		CC	37 (17.13)	40 (17.54)	
rs2737190	5′-UTR	AA	95 (43.98)	73 (32.02)	0.025
		AG	87 (40.28)	118 (51.75)	
		GG	34 (15.74)	37 (16.23)	
rs10116253	5′-UTR	TT	98 (45.37)	74 (32.46)	0.019
		CT	87 (14.35)	116 (50.88)	
		CC	31 (40.28)	38 (16.67)	
rs1927914	5′-UTR	TT	94 (43.52)	74 (32.46)	0.034
		CT	87 (40.28)	118 (51.75)	
		CC	35 (16.20)	36 (15.79)	
rs10759932	5′-UTR	TT	130 (60.19)	110 (48.25)	0.040
		CT	70 (32.41)	98 (42.98)	
		CC	16 (7.41)	20 (8.77)	
rs1927911	Intron	CC	92 (42.59)	72 (31.58)	0.040
		CT	90 (41.67)	120 (52.63)	
		TT	34 (15.74)	36 (15.79)	
rs12377632	Intron	CC	91 (42.13)	78 (34.21)	0.202
		CT	90 (41.67)	112 (49.12)	
		TT	35 (16.20)	38 (16.67)	
rs2149356	Intron	CC	93 (43.06)	77 (33.77)	0.119
		AC	89 (41.20)	113 (49.56)	
		AA	34 (15.74)	38 (16.67)	
rs11536889	3′-UTR	GG	123 (56.94)	123 (53.95)	0.518
		CG	76 (35.19)	91 (33.91)	
		CC	17 (7.87)	14 (6.14)	
rs7037117	3′-UTR	AA	122 (56.48)	123 (53.95)	0.739
		AG	74 (34.26)	86 (37.72)	
		GG	20 (9.26)	19 (8.33)	

*P* value was calculated by a χ^2^–test 3×2 contingency table (df  = 2).

Table 3 presented the association between TLR4 variants and hepatocellular carcinoma risk. Individuals carrying the heterozygous genotypes for the rs10759930, rs2737190, rs10116253, rs1927914, rs12377632 and rs1927911 were associated significantly with decreased risk of hepatocellular carcinoma comparing with those carrying wild-type homozygous genotypes (adjusted odds ratio [OR] by sex and age, from 0.527 to 0.578, *P*<0.01).

**Table 3:** Association between hepatocellular carcinoma and TLR4 single nucleotide polymorphisms.

SNP	Genotype	Odd Ratio (95% CI)	*P* Value	Odd Ratio (95% CI) †	*P* Value
rs10759930	TT	1		1	
	CT	0.587 (0.387–0.889)	0.012	0.550 (0.360–0.839)	0.006
	CC	0.719 (0.417–1.241)	0.237	0.660 (0.379–1.148)	0.141
rs2737190	AA	1		1	
	AG	0.567 (0.375–0.855)	0.007	0.529 (0.348–0.805)	0.003
	GG	0.706 (0.405–1.232)	0.220	0.626 (0.354–1.106)	0.107
rs10116253	TT	1		1	
	CT	0.566 (0.376–0.854)	0.007	0.527 (0.347–0.800)	0.003
	CC	0.616 (0.351–1.081)	0.091	0.553 (0.312–0.981)	0.043
rs1927914	TT	1		1	
	CT	0.580 (0.385–0.876)	0.010	0.547 (0.360–0.830)	0.005
	CC	0.765 (0.439–1.335)	0.346	0.684 (0.387–1.207)	0.190
rs10759932	TT	1		1	
	CT	0.604 (0.406–0.900)	0.013	0.578 (0.386–0.865)	0.008
	CC	0.677 (0.335–1.370)	0.278	0.609 (0.298–1.246)	0.175
rs1927911	CC	1		1	
	CT	0.587 (0.389–0.886)	0.011	0.544 (0.358–0.829)	0.005
	TT	0.739 (0.422–1.295)	0.291	0.661 (0.373–1.172)	0.157
rs12377632	CC	1		1	
	CT	0.689 (0.457–1.038)	0.075	0.651 (0.429–0.987)	0.043
	TT	0.789 (0.456–1.368)	0.399	0.719 (0.411–1.258)	0.248
rs2149356	CC	1		1	
	AC	0.652 (0.433–0.983)	0.041	0.607 (0.400–0.921)	0.019
	AA	0.741 (0.426–1.287)	0.287	0.655 (0.378–1.168)	0.155
rs11536889	GG	1		1	
	CG	0.835 (0.563–1.238)	0.370	0.848 (0.570–1.260)	0.413
	CC	1.214 (0.573–2.571)	0.612	1.336 (0.626–2.851)	0.454
rs7037117	AA	1		1	
	AG	0.868 (0.582–1.293)	0.485	0.842 (0.563–1.259)	0.402
	GG	1.061 (0.540–2.086)	0.863	1.019 (0.516–2.013)	0.957

OR, odds ratio; 95% CI, 95% confidence interval;

† Adjusted for age and sex. *P* value was calculated by a χ^2^–test 2×2 contingency table (df  = 1).

**Figure 1:**
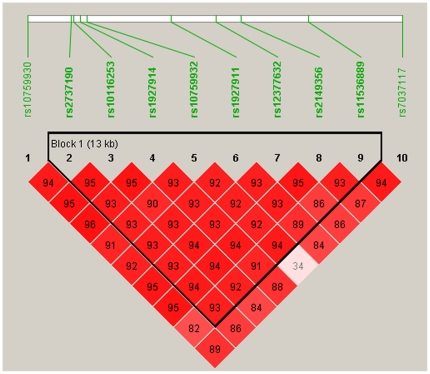
Linkage disequilibrium plot of ten SNPs of the TLR4 gene in patients with hepatocellular carcinoma. D' corresponding to each SNP pair are expressed as a percentage and shown within the respective square. Higher D' is indicated by a brighter red.

Eight of ten TLR4 SNPs were located in 1 haplotype block, and the magnitude of LD between each SNP was extremely high, with pair-wise D'>0.9 (Figure 1). Furthermore, the haplotype analysis was performed for evaluating the frequencies of haplotypes based on the 8 polymorphisms within the block, trying to derive haplotypes specifically correlated with the risk of hepatocellular carcinoma. Four common TLR4 haplotypes (frequency >1%) were found with the accumulated frequency of 90.03% in controls and of 90.52% in cases (Table 4). The distribution of haplotypes between cases and controls was not significant (*P*global  = 0.061). The haplotype GCCCTTAG was associated significantly with decrease of the risk of the occurrence of hepatocellular carcinoma (OR, 0.556, 95% CI, 0.407–0.758, *P* = 0.048) when compared with the most common haplotype ATTTCCCG.

**Table 4:** Association of haplotypes in TLR4 with hepatocellular carcinoma.

Haplotype	Control Frequency†	Case Frequency†	OR (95% CI)	*P*_global_	*P* Value
				0.061	
ATTTCCCG	35.3	30.7	1.042 (0.788–1.378)		0.772
ATTTCCCC	22.5	21.6	0.921 (0.670–1.265)		0.611
GCCCTTAG	20.2	28.3	0.556 (0.407–0.758)		0.000
GCCTTTAG	11.9	9.9	1.083 (0.708–1.655)		0.713

OR, odds ratio; 95% CI, 95% confidence interval;

† , proportion of indicated haplotype (%). *P* value was calculated by a χ^2^–test 2×2 contingency table (df  = 1).

Table 5 shows FPRP estimates for selected results of seven SNPs and one haplotype of TLR4. The association for six TLR4 SNPs showed a FPRP below 0.200, which suggested that thess associations are unlikely to represent a false-positive result. For associations with TLR4 haplotype, the association also yielded a FPRP below 0.200.

**Table 5:** FPRPs for the selected associations between genetic polymorphisms and hepatocellular carcinoma.

SNP or	Odd Ratio	Observed *P* value	Prior probability			
Haplotype	(95% CI)		0.25	0.1	0.01	0.001
SNP						
rs10759930	0.587 (0.387–0.889)	0.012	0.0666	0.1762	0.7018	0.9596
rs2737190	0.567 (0.375–0.855)	0.007	0.0391	0.1087	0.5730	0.9312
rs10116253	0.566 (0.376–0.854)	0.007	0.0386	0.1074	0.5697	0.9304
rs1927914	0.580 (0.385–0.876)	0.010	0.0546	0.1476	0.6557	0.9505
rs10759932	0.604 (0.406–0.900)	0.013	0.0735	0.1922	0.7236	0.9635
rs1927911	0.587 (0.389–0.886)	0.011	0.0630	0.1678	0.6893	0.9572
rs2149356	0.652 (0.433–0.983)	0.041	0.1981	0.4256	0.8907	0.9880
Haplotype						
GCCCTTAG	0.556 (0.407–0.758)	0.000	0.0012	0.0037	0.0391	0.2910

OR, odds ratio; 95% CI, 95% confidence interval.

## Discussion

There is growing evidence that TLR4 genetic polymorphisms impact on risk of cancer including gastric cancer [10] and prostate cancer [8]. TLR4 plays a central role in the signaling pathways that control the innate immune response in response to viral or bacterial infection. TLR4 was up-regulated in the hepatocytes in patients with chronic hepatitis B [17]. Patients with chronic hepatitis B infection will eventually progress into liver cirrhosis and hepatocellular carcinoma [18]. We hypothesized that single-nucleotide polymorphisms of TLR4 are related to the occurrence of hepatocellular carcinoma. In this study, the genetic diversity in TLR4 was examined comprehensively to validate our hypothesis that inherited differences in TLR4 were associated with hepatocellular carcinoma. To our knowledge, this is the first attempt to investigate a series of common SNPs located in the TLR4 gene in patients with hepatocellular carcinoma.

Activation of TLR4 can cause liver injury under several clinical conditions such as hemorrhagic shock [19], hepatic ischemia reperfusion injury [20], alcohol hepatitis [21] and chronic hepatitis B [17]. Experimental investigations revealed that TLR4 gene mutations were associated with low response to viral and bacterial infection and therefore led to a reduction in the innate immune response and inflammation. The missense mutation in the third exon of TLR4 rendered the C3H/HeJ and C57B/10ScCr mice resistant to the toxic effects of LPS from Gram-negative bacteria [22]. TLR4 mutant (C3H/HeJ) mice subjected to trauma had less systemic inflammation and liver injury than wide-type controls [23]. TLR4 mutant mice display natural resistance to acid-induced acute lung injury [24].

Our results in this study suggested that inherited variation in TLR4 influences the risk of hepatocellular carcinoma. Specifically, we firstly described the positive association between four 5′-UTR polymorphism (rs10759930, rs2737190, rs10116253 and rs1927914) and one intron polymorphism (rs1927911) of TLR4 gene and the risk of hepatocellular carcinoma. There is a significantly decreased risk of hepatocellular carcinoma in individuals carrying the heterozygous genotypes for the rs10759930, rs2737190, rs10116253, rs1927914, rs12377632 and rs1927911 when comparing with those carrying wild-type homozygous genotypes (OR, from 0.527 to 0.578, *P*<0.01). Among these positive SNPs in the present study, rs10759930, rs2737190, rs10116253, rs1927914 and rs12377632 were located in 5′-UTR. Cheng et al [25] found that there is a significantly increased risk of prostate cancer in individuals carrying the variant homozygous genotype for rs10759932 located in 5′-UTR when comparing with those carrying wild-type homozygous genotypes. In another study by Chen et al [9], homozygosity for the variant alleles of rs10116253 was associated with a statistically significant lower risk of prostate cancer. Our results suggested that mutation of TLR4 gene in 5′-UTR might reduce the occurrence of hepatocellular carcinoma. Although the observed two-fold decrease in risk is modest, our finding is intriguing because genes in multiple pathways alter the risk for hepatocellular carcinoma, and each individual gene is likely to contribute only a modest risk. The 5′-UTRs polymorphisms of TLR4 presumably affect transcription and/or translation among individuals with hepatocellular carcinoma. As 5′-UTRs influence the translation of regulatory proteins during growth, differentiation, embryonic development, and stress, modulation of 5′-UTR activity plays a role in the development or progress of specific forms of cancer. In most cases, the 5′-UTR is involved in the regulated expression of a key protein concerning with growth or differentiation in normal tissues. A deregulated expression of such proteins could play a role in neoplastic transformation [26]. In our study, the TLR4 SNPs may potentially exert regulator effects and therefore might decrease the risk for hepatocellular carcinoma. Due to the strong regional correlation across TLR4, these SNPs may serve as a proxy for the predisposing variants or the polymorphism themselves may have functional consequences on TLR4 expression or signaling activity. Alteration in TLR4 activity influences innate immunity and inflammation, which in turn may affect hepatocellular carcinoma susceptibility. Functional assays are needed to elucidate the molecular mechanisms underlying these associations. In addition, only individuals carrying heterozygous genotypes have a decrease for the risk of hepatocellular carcinoma development in our study. Such situation is not usual but is observed in some investigations of other cancers, such as esophageal squamous cell carcinoma [27], pancreatic cancer [28], and breast cancer [29], [30]. Gene coding sequences account for only 1.5% of the human genome. Subtotal association studies have largely focused on known coding sequences. However, there are accumulating evidences that mutations in the splice, donor and acceptor sites or enhancer, intron and promoter elements may be important in genetic expression and regulation [31]. Some investigations have found that genetic SNPs within are involved in tumorigenesis, including prostate cancer [32], breast cancer [33] and other cancers [34],[35]. In the present study, three SNPs (rs1927911), which located in the region of intron of TLR4, appeared to decrease risk of hepatocellular carcinoma (OR, from 0.572 to 0.607, *P*<0.01) significantly also. In the investigation by Chen et al [9], the risk of prostate cancer was lower significantly in individuals carrying variant carriers in rs1927911 (OR, 0.63, 95% CI, 0.41–0.95).

LD analysis showed that the eight TLR4 SNPs were in high LD and located in one haplotype block. Individuals carrying haplotype GCCCTTAG may decrease the risk of hepatocellular carcinoma significantly (OR, 0.556, 95% CI, 0. 0.407–0.758, *P* = 0.000) compared to those who carried the most common haplotype ATTTCCCG. These results indicate that the TLR4 SNPs commonly linked to the GCCCTTAG haplotype are likely to be protective.

In conclusion, our study provided evidence of a close association between TLR4 sequence variants and hepatocellular carcinoma. Although the contribution of the SNPs in TLR4 is modest, these sequence variations, together with haplotype, may define a genetic susceptibility background for hepatocellular carcinoma, suggesting that TLR4 gene variation may play an important protective role in the occurrence of hepatocellular carcinoma. More studies are needed to validate this finding in independent populations and to understand the mechanism by which TLR4 sequence variants affect the pathological role of TLR4 in the signaling pathways that control carcinogenesis.

## Materials and Methods

### Case-control study cohorts

In total, this case-control study consisted of 216 patients with hepatocellular carcinoma (hepatocellular carcinoma) from Ruijin Hospital, Shanghai Jiaotong University Medical School were enrolled and 228 non cancer control. All patients and controls were unrelated Han Chinese and gave informed consent. The study protocol was approved by the independent ethics committee of Ruijin Hospital, Shanghai Jiaotong University Medical School. The content was written. The case study patients were enrolled consecutively from June 2008 to June 2010 at Ruijin Hospital, Shanghai Jiaotong University Medical School. All cases were newly diagnosed, previously untreated (chemotherapy or transcatheter arterial chemoembolization, TACE). HCC was diagnosed by the elevation of alpha-fetoprotein (>400 ng/ml) or by pathological examination in combination with the results of examination of iconography including computer tomography (CT) and magnetic resonance imaging (MRI). Clinical classification was dependent of the results of CT or MRI according to International Union Against Cancer (UICC) tumor-node-metastasis (TNM) staging system [36]. The patients with hepatocellular carcinoma were proved not to have other kinds of cancer CT, MRI or positron emission computed tomography (PET-CT). In addition, these subjects included in this investigation were negative for antibodies to hepatitis C virus, hepatitis D virus and had no other types of liver disease (for example, autoimmune hepatitis, toxic hepatitis, and primary biliary cirrhosis or Budd-Chiari syndrome). The option of treatments for hepatocellular carcinoma includes hepatectomy, liver transplantation, chemotherapy or/and TACE. HBV carriers were defined as positive for both hepatitis B surface antigen and antibody immunoglobulin G to hepatitis B core antigen. In order to reduce the confounding effect of HBV infection in research of genetic susceptibility to HCC, controls were randomly selected from the individuals who attended hepatitis examination in the hospital during the period of case collection. The selection criteria for the controls included also no individual history and no diagnosis of any cancer at the time of ascertainment and frequency matching to the patients on age and gender.

### Selection of TLR4 Single-Nucleotide Polymorphisms

The subsets of reported TLR4 SNPs were determined by using publicly available genotype data from the International HapMap project (http://hapmap.ncbi.nlm.nih.gov/index.html.zh). We downloaded the data for TLR4 SNPs that spanned ∼2 kb upstream of the TLR4 transcription start site and ∼1 kb downstream of the 3′-untranslated region (UTR). Ten SNPs were selected by using the criteria of minor allele frequencies (MAF) ≥5% in Chinese people population, including the predicted 5′- UTR, the introns and the predicted 3′- UTR. An important goal in this study was to evaluate common haplotypes of TLR4 sequence variants with use of a limited number of TLR4 SNPs. SNPs located in exons and promoter regions were excluded from the study because MAF of all these SNPs was lower than 5% in Chinese people population.

### Genotyping

Genomic DNA was extracted from peripheral blood leukocytes. The genomic regions of interest were amplified by multiplex polymerase chain reactions (PCRs). These PCR reactions were performed in a total volume of 10 µl containing 20 ng DNA, 3 µ mol dNTPs, 1X PCR Gold buffer with 28 µ mol MgCl_2_, 4 µ mol of each primer and 0.5 unit of Hotstar HiFidelity DNA Polymerase (Qiagen,German). Samples were denatured at 95°C for 15 min followed by 15 cycles of 94°C for 40 s, 63°C for 60 s, followed by 72°C for 1.5 min, followed by 20 cycles of 94°C for 40 s, 56°C for 40 s, followed by 72°C for 1.5 min, and a final extension at 72°C for 8 min.

All ten SNPs were genotyped by SNaPshot Multiplex kit (Applied Biosystems, Foster City, CA, USA). In brief, 15 µl of mixed PCR products from the above reactions were then incubated with 2 U of shrimp alkaline phosphatase (SAP) and 2 U of exonuclease I/(Exo I) at 37°C overnight, followed by heating at 75°C for 15 min to inactivate SAP and Exo I. Following this, 10 µl of reaction mix included 5 µl of SNaPshot reaction mix, 3 µl of pooled PCR products. 1 µl of pooled SNaPshot primers and 1 µl of deionized water were incubated in a GeneAmp 9600 thermal cycler by 25 cycles at 96°C for 10 s, 50°C for 5 s, and 60°C for 30 s, and finally 60°C for 30 s. Then, 1 U of SAP and 1 U CIP (calf intestinal phosphatase) was added to SNaPshot product and incubated at 37°C for an hour to deactivate the enzyme. SNaPshot product was diluted for 20 times. The final reaction mix containing 8.6 µl of Hi-Di Formamide, 0.5 µl of SNaPshot product and 0.9 µl of GeneScan-120 LIZ internal size standard (Applied Biosystems) was denatured at 95°C 5 min. Samples were placed at −20°C for storage prior to electrophoresis. The genotypes of 13 SNPs were identified by different fluorescent signals by ABI-3730XL Genetic Analyzer (Applied Biosystems). The conditions of electrophoresis were according to the guildlines by Applied Biosystems. The data were analyzed by the software of GeneMapper 4.0.

Genotype analysis was performed in a blinded manner so that the staff was unaware of the cases or control status. For quality control, a 10% masked random sample of cases and controls was tested repetitively by different investigators and all the results were completely concordant.

### Statistical Analysis

Data were expressed as proportions or mean and standard deviation (SD). All these tests were performed with the SPSS 13.0 version for Windows. All reported *P* values were two sided. Grouped data were compared by the Mann-Whitney *U* test.

The Hardy-Weinberg equilibrium (HWE) test using two-sided χ^2^ analysis was done for each SNP among cases and controls. Genotype frequency differences were tested between hepatocellular carcinoma group and control group were tested for each SNP by two-sided χ^2^ test with 2 degrees of freedom.

Odds ratios (ORs) of hepatocellular carcinoma for the variant-allele carriers (homozygous and heterozygous) versus homozygous wild-type allele carriers were estimated by unconditional logistic regression and adjusted for age (≤55 or >55).

Haplotype block structure and the estimates of pair-wise linkage disequilibrium (LD) (D') were determined by using Haploview software (http://www.broadinstitute.org/haploview/haploview/index.php). Haplotype frequency was estimated with the statistical method by implementing the computer program PHASE. A global score test was used to assess the difference in haplotype frequency distributions between cases and controls. Association between the haplotypes and hepatocellular carcinoma was performed with the χ^2^ test.

False-positive report probabilities (FPRPs) for those associations observed to be statistically significant (*P*<0.05) were calculated to account for potential false positives because some associations would arise by chance. FPRP is defined as the probability of no true association between genetic variants and disease given the statistically significant finding. The values of false-positive report probability (FPRP) were assessed by the use of method described by Wacholder et al [37]. We assumed prior probabilities for associations with hepatocellular carcinoma status under a dominant model of 0.1 for each SNP and 0.01 for each haplotype. FPRP <0.200 was considered to indicate a noteworthy association.
